# Offering healthier food options

**DOI:** 10.1038/s43856-021-00041-z

**Published:** 2021-10-13

**Authors:** Katharine Barnes

**Affiliations:** Communications Medicine, https://www.nature.com/commsmed

## Abstract

Reducing energy intake is known to result in weight loss. However, there is a need for real-world data on the impact of specific interventions to reduce energy intake. A recent trial in *PLOS Medicine* evaluated whether reductions in portion size and in the availability of high energy foods in workplace cafeterias could reduce energy consumption.

**Figure:**
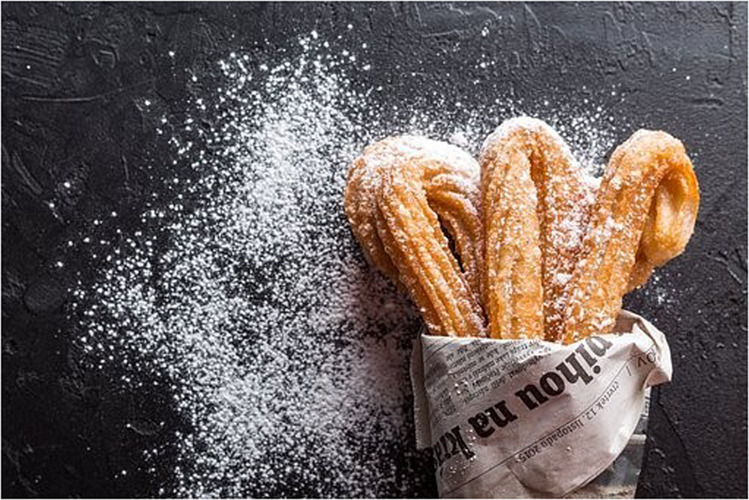
Pixabay

The second most common place most adults consume food is at work, where they consume up to a fifth of their energy intake. It has been calculated that just a small reduction in calories consumed per person per day could prevent further weight gain.

Reynolds and colleagues^1^ undertook a stepped-wedge randomised controlled trial over a 25-week period across 19 workplace cafeterias serving over 20,000 predominantly manual workers. The cafeterias were typically in remote locations with no local food outlets to purchase food or drink. Following a baseline period, during which no intervention was implemented, high energy products were replaced with low energy products. Later, a reduction in portion size of at least 10% was introduced for all the remaining high energy products. The weeks in which the interventions were introduced were randomly allocated across different canteens. Posters and internal communications were circulated describing a new “health initiative”, without inclusion of specific details of what this entailed.

The primary outcome being assessed was the total energy (kcal) per day purchased from the food categories that had been modified. Total energy purchased decreased significantly when availability was reduced, and decreased even further when both availability and portion size were reduced. The secondary outcomes included energy (kcal) per day purchased from the food categories that had not been altered and total energy purchased per day, both of which also reduced.

Whilst purchase of food was used as a proxy for energy consumed, it is known that people usually consume approximately 90% of food that they select in such settings, suggesting this data represents a genuine reduction in energy consumption. Changes such as those trialled in these cafeterias could potentially also be easily implemented in schools and universities, forming part of broader strategies to tackle obesity.
